# Fish Gut Microbiome Analysis Provides Insight into Differences in Physiology and Behavior of Invasive Nile Tilapia and Indigenous Fish in a Large Subtropical River in China

**DOI:** 10.3390/ani13152413

**Published:** 2023-07-26

**Authors:** Yaqiu Liu, Chunni Kou, Yuefei Li, Jie Li, Shuli Zhu

**Affiliations:** 1 Pearl River Fisheries Research Institute, Chinese Academy of Fishery Sciences, Guangzhou 510380, China; 2 Guangzhou Scientific Observing and Experimental Station of National Fisheries Resources and Environment, Guangzhou 510380, China; 3 Key Laboratory of Aquatic Animal Immune Technology of Guangdong Province, Guangzhou 510380, China

**Keywords:** gut microbiome, Nile tilapia, invasive, black Amur bream, physiology, Pearl River

## Abstract

**Simple Summary:**

The Nile tilapia (*Oreochromis niloticus*) (NT) is an aggressive and omnivorous species that competes with native fishes for food resources, and it has successfully invaded much of the Pearl River basin in China. Intensive research has focused on the gut microbiota of Nile tilapia in artificial culture. However, differences in gut microbes between invasive Nile tilapia and indigenous fish are relatively less well characterized. In the present study, we chose the black Amur bream (*Megalobrama terminalis*) as a native omnivorous fish that shared a common habitat and partially overlapped resource usage with Nile tilapia. Here, we investigated the gut microbiomes of invasive Nile tilapia and indigenous black Amur bream (BA) in the same river section using high-throughput 16S rRNA gene sequencing. The results indicated that the gut microbiome of NT had several special characteristics, e.g., higher alpha diversity and greater niche breadth, compared with the bream. Thus, the aim of the present study was to investigate the role of gut microbiomes between an indigenous fish (black Amur bream) and invasive Nile tilapia from the low region of the Pearl River basin to better understand the microbial complexity and diversity of their gut microbiota.

**Abstract:**

The gut microbiome is thought to play vital roles in host fitness and local adaptation to new environments, thereby facilitating the invasion of the host species. The Nile tilapia (*Oreochromis niloticus*) (NT) is an aggressive and omnivorous species that competes with native fishes for food resources, and it has successfully invaded much of the Pearl River basin in China. Here, we investigated the gut microbiomes of invasive Nile tilapia and indigenous black Amur bream (BA) in the same river section using high-throughput 16S rRNA gene sequencing. The results indicated that the gut microbiome of NT had several special characteristics, e.g., higher alpha diversity and greater niche breadth, compared with the bream. The gut microbiota of the small size of Nile tilapia (NTS) and small size of black Amur bream (BAS) groups were dominated by Proteobacteria, while those of the NTS and large size of Nile tilapia (NTL) and BAS and large size of black Amur bream (BAL). BAL and NTL were characterized by Firmicutes and Fusobacteriota, respectively. We found that *Pseudomonas*, *Cetobacterium*, *Ralstonia*, and *Romboutsia* were biomarkers of the NTS, NTL, BAS, and BAL groups, respectively. Moreover, the results collectively suggested that the clustering coefficients of BAL and NTL networks were greater than those of BAS and NTS networks, and BAS had the smallest network among the four groups. Positive interactions between two ASVs dominated the BAS, NTS, and NTL networks, while the proportion of negative interactions between two ASVs in the BAL network was remarkably increased. Low levels of interspecies competition in the NT gut microbiome would contribute to high diversity in the dietary niches and would also benefit the survival and local adaptation of the host. Our results identified specific biomarkers of gut microbial species in invasive Nile tilapia and provided useful information concerning how to monitor and manage invasive Nile tilapia populations.

## 1. Introduction

Nile tilapia (*Oreochromis niloticus*) is a fish native to Africa that has been introduced as an economically important species in aquaculture. In southern China, Nile tilapia have escaped captivity and successfully invaded much of the Pearl River basin; the species now dominates these aquatic communities, especially in the major rivers of Guangdong Province [1]. It has been reported that Nile tilapia is aggressive, has omnivorous feeding habits, and can effectively compete with native fishes for food resources [2,3]. Nile tilapia populations that have been successfully established can outcompete native species for food and habitat space, leading to the decline of native fish populations and potentially irreversible ecological changes in the Pearl River basin [1,4]. Related studies have indicated that the feeding and excretion habits of tilapia result in increased turbidity in aquatic habitats (Gu et al., 2015) [1,5]. Furthermore, the variation in turbidity of the habitats can alter total nitrogen (N) and phosphorus (P) and affect periphyton and phytoplankton biomass in freshwater ecosystems [6,7,8]. Therefore, understanding the feeding behavior of invasive and native fishes will provide insights into controlling Nile tilapia populations in the Pearl River basin.

One approach for analyzing fish feeding behavior is to examine the gut microbiome [9,10,11]. Many studies have indicated that microbiomes play essential roles in host fitness and local adaptation to new environments [9,11]. Notably, the digestion of materials and nutrient uptake of animal species are strongly affected by the vast genetic repertoire of gut microbiota [12,13,14]. A high proportion of Cyanobacteria was found in the gut microbiome of invasive silver carp in the Mississippi River Basin, indicating that their major food source was green algae) [11,15]. Variation in the availability of food resources is the most direct factor modulating the composition of intestinal microbial communities [16,17]. Nile tilapia feed on phytoplankton, macrophytes, insects, detritus, and zooplankton depending on the life stage [18]. Moreover, various life stages of this species show differences in feeding habits. Changes in feeding habits may be a potential driving force in the composition of the gut microbial community in Nile tilapia.

Intensive research has focused on the gut microbiota of Nile tilapia in artificial culture [19,20,21]. However, differences in gut microbes between invasive Nile tilapia and indigenous fish are relatively less well characterized. In the present study, we chose the black Amur bream (*Megalobrama terminalis*) as a native omnivorous fish that shared a common habitat and partially overlapped resource usage with Nile tilapia. Due to the continuous enhancement of human activities (e.g., water conservancy projects, alien species invasion, waterway dredging, water pollution, and overfishing), a decline in wild *M. terminalis* populations has occurred in the Pearl River basin during the past decade [22,23]. Thus, the present study aimed to investigate the role of gut microbiomes between an indigenous fish (black Amur bream) and invasive Nile tilapia from the low region of the Pearl River basin to better understand the microbial complexity and diversity of their gut microbiota. Fish gut microbiota and the feces discharged into the water can reflect fish diet preferences, physiological status, and presence in the river [15]. In addition, understanding the microbial community in the fish guts of Nile tilapia and native fish can provide useful information concerning how to monitor and manage Nile tilapia populations by exploring specific biomarkers based on gut microbial species.

## 2. Materials and Methods

### 2.1. Fish Sample Collection

**Figure 1:**
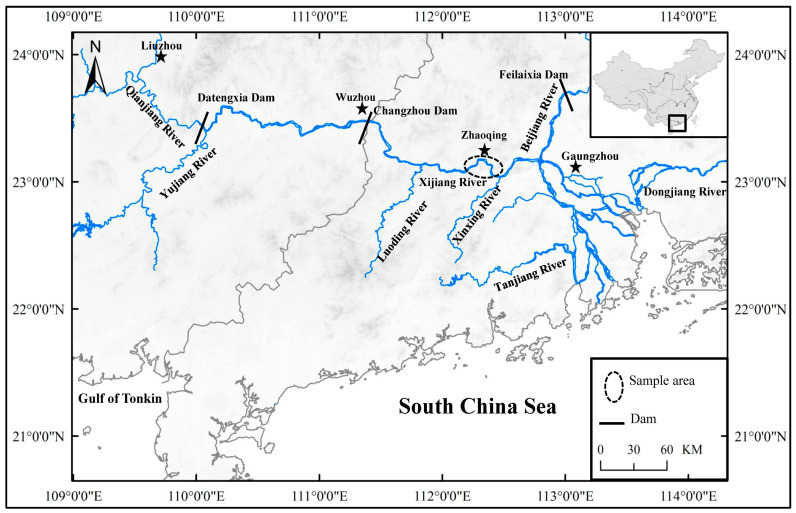
Locations of the sample area. Note: stars was shown the location of the city.

This study was conducted in the Zhaoqing section of the Pearl River, located in Guangdong Province, China (latitude 23°02′–04′ N, longitude 112°25′–31′ E) (Figure 1). Sampling took place in August 2021, using circular cast nets with a diameter of 15 m and a mesh size of 4 cm. Table S1 provides basic environmental information about the sample sites. The length of the fish’s body (BL) was measured from the tip of the mandible to the base of the caudal fin, with a precision of 1 mm. The body weight (BW) was measured with a precision of 1 g. For each fish, we collected gut contents of Nile tilapia (NT) and black Amur bream (BA): small size (BL: 140–160 mm) of Nile tilapia (NTS) (*n* = 8) and large size (BL: 240–260 mm) of Nile tilapia (NTL) (*n* = 11), and small size (BL: 140–160 mm) of black Amur bream (BAS) (*n* = 6)and large size (BL: 240–260 mm) of black Amur bream (BAL) (*n* = 8). To investigate the fish gut microbiome, prior to dissection, the fish were euthanized with an overdose of MS 222 (3-aminobenzoic acid ethyl ester methane sulfonate, Sigma, Darmstadt, Germany). In order to prevent contamination from the fish’s skin and remove transient bacteria, aseptic instruments were used to dissect the entire intestinal tract of each individual fish sample. The dissected intestines were promptly rinsed in a solution of 75% ethanol and sterile water. The gut contents, which were utilized for DNA extraction, were immediately submerged in liquid nitrogen and then transferred to an ultra-low-temperature freezer, where they were stored at −80 °C until further analysis.

### 2.2. DNA Extraction and Amplification

Each sample, weighing approximately 0.2 g, was subjected to DNA extraction using the QIAamp DNA Stool Mini Kit (Qiagen, Valencia, CA, USA). The extracted DNA samples were stored at −80 °C until further use. For the amplification of the V3–V4 hypervariable region of the bacterial 16S rRNA gene, specific primer pairs 341F (5′–CCTAYGGGRBGCASCAG–3′) and 806R (5′–GGACTACNNGGGTATCTAAT–3′) were used. The PCR amplification was carried out using an ABI GeneAmp^®^ 9700 PCR thermocycler (ABI, CA, USA). Subsequently, the total DNA extracted from the fish gut samples was sent to Majorbio Bio-Pharm Technology Co., Ltd., located in Shanghai, China, for further analysis using Illumina MiSeq sequencing.

### 2.3. Bioinformatic and Statistical Analysis

Following demultiplexing, the obtained sequences underwent quality filtering using fastp and were merged using FLASH (v1.2.11) [24]. Subsequently, the high-quality sequences were de-noised using the DADA2 plugin in the Qiime2 pipeline (version 2020.2) with recommended parameters. This de-noising process, based on error profiles within samples, allows for single nucleotide resolution [25,26]. The sequences that underwent denoising using DADA2 are commonly referred to as “amplicon sequence variants” (ASVs). To mitigate the impact of sequencing depth on alpha and beta diversity calculations, the number of sequences from each sample was rarefied to 20,000, which resulted in an average Good’s coverage of 97.90%. Taxonomic classification of the ASVs was carried out using the RDP classifier within the Qiime2 platform, utilizing the Silva 16S rRNA database (v138). The metagenomic function was predicted using PICRUSt2 (Phylogenetic Investigation of Communities by Reconstruction of Unobserved States) with ASV representative sequences [27]. Alpha diversity indices, including Ace, Chao1 richness, Shannon’s index, and the PD tree, were computed using Mothur v1.30.1 [28]. The similarity between microbial communities in different samples was assessed using non-metric multidimensional scaling (NMDS) analysis based on Bray-Curtis dissimilarity, performed using the vegan R package. To evaluate the significance of differences between groups, an ANOSIM test was conducted using the vegan package. Additionally, a linear discriminant analysis (LDA) effect size (LEfSe) was applied to identify bacterial taxa (from phylum to genus level) that exhibited significant abundance differences among the various groups. The MetaStat method was employed to compare species abundance between the BA and NT groups and select species with significant differences. A random forest supervised machine learning model was applied to identify the importance of species with significant differences between BA and NT groups [29], and the model was verified by ROC (receiver operating characteristic curve) analysis [30]. Further indicator species analysis was used to identify biomarkers for different groups. Ecological network analysis was used to explore bacterial interactions in the gut microbiota of different groups. The modules within each ecological network were delineated using the fast-greedy modularity optimization method [31]. The resulting networks were visualized using Cytoscape (v3.8.0) software. A one-way analysis of variance (ANOVA) in SPSS Statistics 28.0 was used to evaluate the differences in alpha diversity, niche breadth, and mean proportion of metabolic pathways among groups. A *p*-value below 0.05 was used to determine statistical significance.

### 2.4. Habitat Niche Breadth Analysis

The bacterial communities in the gut are influenced by the trait of niche breadth, which has been demonstrated by studies [32,33]. Species with a broader niche breadth, known as generalists, are expected to exhibit greater metabolic flexibility and long-term persistence at the regional community level over time. On the other hand, specialists have a narrower niche and are more restricted to specific habitats and times [34,35]. In this study, the generalists and specialists within the gut bacterial communities across different groups were identified using Levins’ niche breadth index.

$$B_{i}=\frac{1}{\sum_{i=1}^{r}{\left(P_{ij}\right)}^{2}}$$

Note: The niche breadth of ASV_j_ in a community is denoted by the variable *B_i_*. The total number of species present within the community is represented by the variable r, while *P_ij_* represents the relative abundance of ASV_j_ in community *i* [33]. Based on a threshold of *B_i_* > 3 or *B_i_* < 1.5, each taxon was classified as either a specialist or a generalist. Furthermore, to measure the habitat niche breadth of the gut bacterial community, we calculated the average *B_i_* values across all taxa [33].

## 3. Results

### 3.1. Differences between Invasive and Indigenous Fish in Gut Microbiome Composition

**Figure 2:**
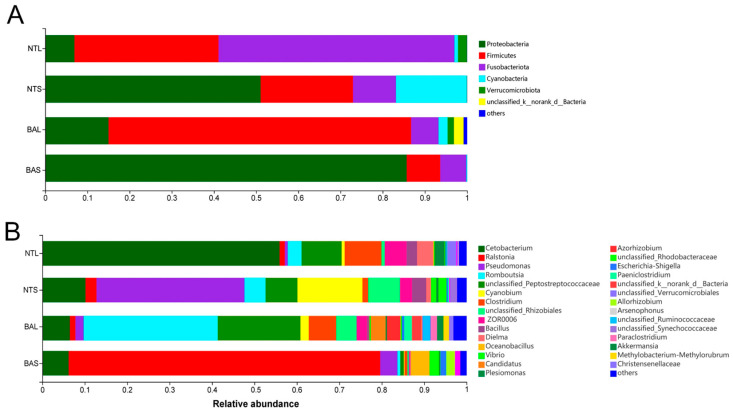
Dominant gut microbiota composition in the different groups at phylum (**A**) and genus (**B**) level; each bar represents the average relative abundance of each bacterial taxon within a group at the phylum and genus level.

**Figure 3:**
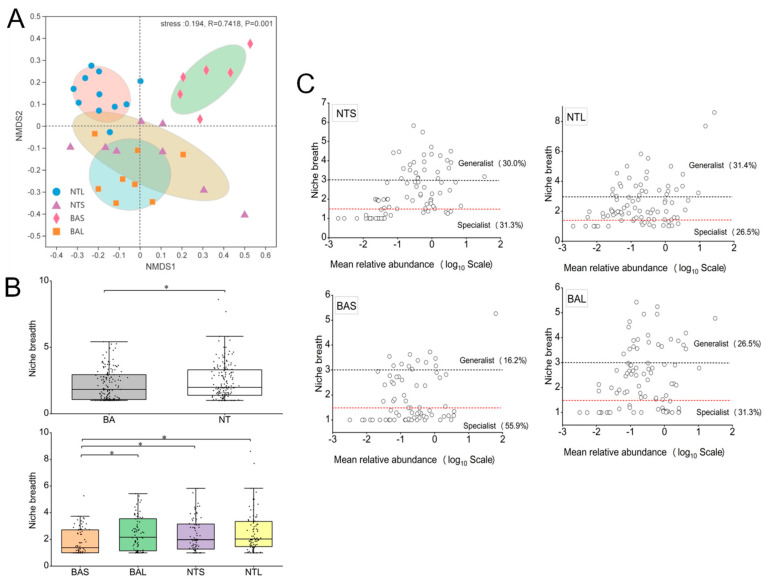
An overview of the data. (**A**) NMDS (non-metric multidimensional scaling) plot showing the microbial community differences among all the samples based on Bray-Curtis distance. The individual sample is color-coordinated according to the different fish gut groups; significance tests of the bacterial community composition with analysis of similarities (ANOSIM), indicating the significance of groups based on Bray-Curtis distances; (**B**) Boxplots illustrating mean habitat niche breadth from all taxa in each sample of bacterial. * Means a significant difference between two populations (*p* < 0.05); (**C**) The mean abundances of ASVs of Bacteria versus habitat niche breadth among different groups.

The microbial complexity in NT and BA was estimated based on alpha diversity (Shannon, PD tree, Chao1, and Ace indices), and the results showed distinct differences (Table 1). The Shannon indices of the BAL, NTS, and NTL groups were greater than those of the BAS group, whereas there were no significant differences among the BAL, NTS, and NTL groups. The Chao1, PD tree, and Ace indices of the four groups showed the same results as the Shannon index. Figure 2A illustrates the abundance of major phyla commonly observed in all four groups. The gut bacteria of NTS and BAS were dominated by Proteobacteria (51.1–85.7%), while those of BAL and NTL were characterized by Firmicutes (71.7%) and Fusobacteriota (55.9%), respectively. At the genus level, the most abundant bacterial taxa in each fish gut group were observed at the genus level (Figure 2B). *Cetobacterium* was the most abundant genus in the NTL group, whereas the most abundant genus in the BAS group was *Ralstonia* (Figure 2B). *Pseudomonas* was the dominant genus of the NTS group, while the dominant genus of the BAL group was *Romboutsia*. To investigate the gut microbiomes in different fish gut samples, an NMDS plot was used to compare the similarity in the microbial community compositions of the fish gut samples (Figure 3A). The BAS samples formed a cluster and were distinctly separated from the clusters of BAL, NTS, and NTL samples (ANOSIM analysis: *R* = 0.742, *p* = 0.001; Adonis analysis: *R*^2^ = 0.332, *p* =0.001). The clustering pattern indicated that samples from NTS were closer to the NTL samples than BAS samples were to the BAL samples and that NTS samples were closer to the BAL samples than to the NTL samples.

### 3.2. Partitioning the Gut Microbial Community into Generalists and Specialists

The average community niche breadth for gut bacterial communities in BA was significantly lower than for NT (Figure 3B). BAS had the narrowest niche breadth among the four groups (Figure 3B). Furthermore, BAS and BAL specialists occupied 55.9% and 31.3% of the total diversity, respectively, higher than the corresponding values in NTL and NTS, where they accounted for 26.5% and 31.3%, respectively (Figure 3C). By contrast, the proportions of generalists in NTL and NTS were higher than in BAS and BAL.

### 3.3. Microbial Populations in Response to Fish Interspecific and Intraspecific Differences

**Figure 4:**
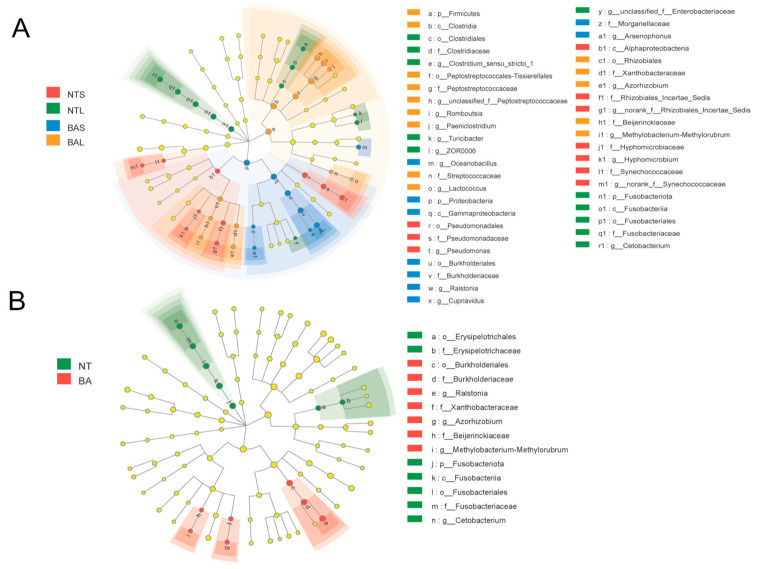
LEfSe identified the most differentially abundant taxons among different species (**A**) and groups (**B**). The dots in the center showed the ASVs at the phylum level, whereas the outer circle of dots showed the ASVs at the genus level. The colors of the dots and sectors present the most abundant ASVs in the different groups, respectively. The yellow color indicates ASVs that showed similar abundance in all compartments. The colored sectors give information on phylum (full name in the outermost circle, given only for phylum showing significant differences between groups, class, order, family, and genus that were significantly different between groups are shown on the right side of the figure.

**Figure 5:**
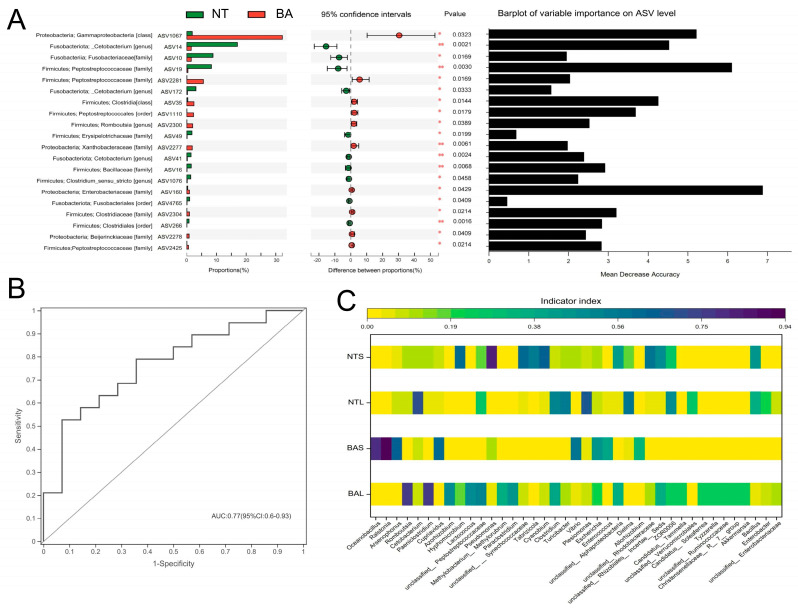
An overview of the data. (**A**) An extended error bar plot showed the ASVs that have significantly different abundances in the guts of NT and BA. Only ASVs with mean proportions larger than 0.5% in the two groups of samples were shown. Bar plot of the AUC of PRC for random forest classifiers for various discriminative ASVs of the fish gut microbiome. * Means significant difference between two populations (*p* < 0.05); ** Means very significant difference between two populations (*p* < 0.01). (**B**) The AUC of ROC for random forest models showed a strong performance of models in classifying NT and BA. (**C**) A heatmap presented the indicator index among four different groups.

Linear discriminant analysis effect size (LEfSe) was employed to characterize the microbial communities and detect significant variations in the abundance of different fish groups (Figure 4). The results showed that the genera *Cetobacterium* and *Clostridum_sensu_stricto_1* were significantly different in NTL samples compared with the other groups. The abundance of the genus *Pseudomonas* was significantly higher in the NTS group than in other groups. The BAS group showed significantly higher abundances of the genera *Oceanobacillus*, *Ralstonia*, *Arsenophonus*, and *Cupriavidus* compared with the other groups (Figure 4A). The BAL group showed significantly higher abundances of the genera *Paeniclostridium* and *Romboutsia* (Figure 4A). Meanwhile, Figure 4B indicated that there were significant differences in family Fusobacteriaceae, Erysipelotrichceae Burkholderiaceae Xanthobacteraceae, Beljerinckiaceae. A Wilcoxon rank-sum test identified 20 ASVs that were significantly different in abundance between the BA and NT groups (Figure 5A). Among these, 10 ASVs had a higher abundance in NT, and the remaining ASVs were more abundant in BA. Specifically, ASV14 (*Cetobacterium* genus), ASV10 (Fusobacteriaceae family), and ASV19 (Peptostreptococcaceae family) showed much higher abundance in the NT group than in BA, whereas ASV1067 (Gammaproteobacteria class), ASV35 (Clostridia class), and ASV160 (Enterobacteriaceae family) had significantly higher abundance in BA than in NT. Furthermore, we utilized the random forest classifier, a machine learning algorithm, to assess the predictive capability of ASVs for interspecific differences among fish. This allowed us to evaluate the performance and effectiveness of ASVs in predicting the variations observed between different fish species. ASV160 was the best for discriminating NT and BA groups, and the classification accuracy was calculated based on the area under the curve (AUC) (Figure 5B). Based on indicator species analysis of the four fish gut groups, we found that *Pseudomonas*, *Cetobacterium*, *Ralstonia*, and *Romboutsia* held the highest indicator indices in the NTS, NTL, BAS, and BAL groups, respectively (Figure 5C).

### 3.4. Network Analysis and Topological Roles of the Gut Microbiome among Different Groups

**Figure 6:**
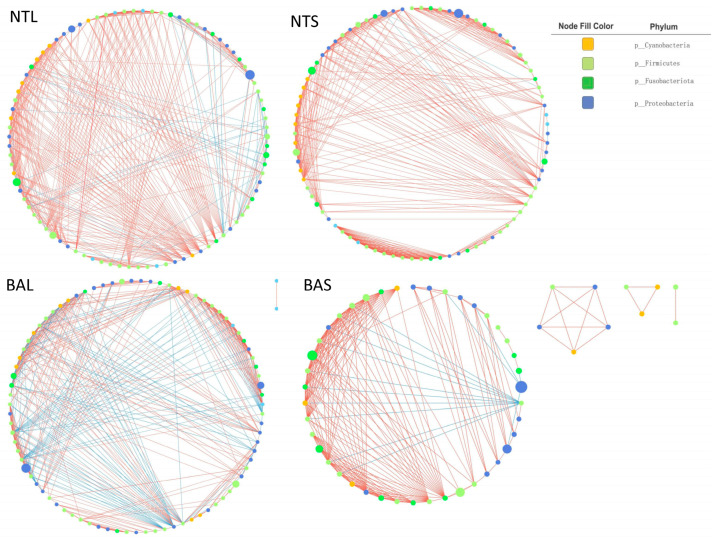
Ecological networks of gut microbiota in the four groups. The analyses were constructed using the top 50 most abundant ASVs of the gut microbial community in the different groups; the ones displayed in the picture were significant at *p* < 0.05 with r ≥ 0.6. Circles represent species; the size of the circle represents abundance. The edges represent the correlation between the two ASVs; the thickness of the edge represents the strength of the correlation; and the color of the line: red represents the positive correlation while blue represents the negative correlation.

We investigated the species interactions of the gut microbiota in the NTS, NTL, BAS, and BAL groups via network analysis. The networks were visualized using Cytoscape software (Figure 6), and the patterns of the four networks were further analyzed and shown in Table 2. The red edges represent positive interactions between two ASVs, and the blue edges represent positive interactions between two ASVs. The BAS network had 52 nodes and 224 edges, and the BAL network consisted of 85 nodes and 429 edges. The NTS network comprised 84 nodes and 422 edges. The NTL network had 87 nodes and 467 edges. In these four networks, more than 80% of the nodes (ASVs) were affiliated with Proteobacteria, Firmicutes, Fusobacteriota, or Cyanobacteria. The results indicated that the clustering coefficients of the BAL and NTL networks were greater than those of the BAS and NTS networks, and BAS had the smallest network among the four groups. Positive interactions between two ASVs dominated the BAS, NTS, and NTL networks, while the proportion of negative interactions between two ASVs in the BAL network was remarkably increased.

### 3.5. Functional Prediction of Gut Microbiome Digestion-Related Bacteria Analysis

**Figure 7:**
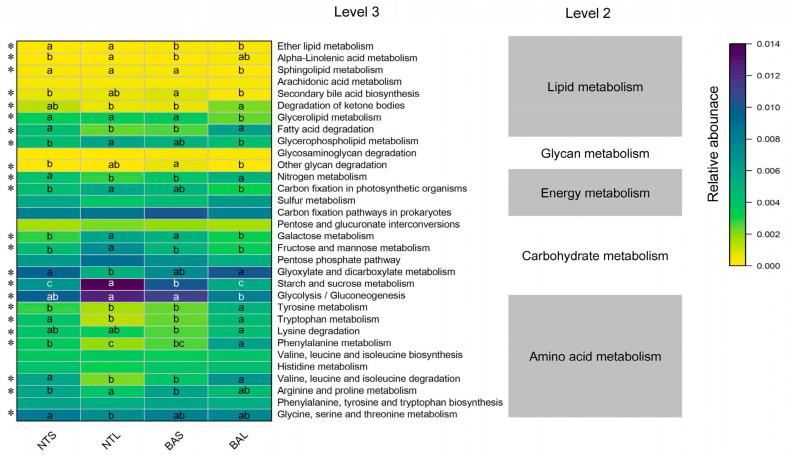
KEGG categories derived from the 16S rRNA sequences of the fish gut microbiomes by PICRUSt2. Heatmap presenting the abundance of digestion-related bacterial gene functions among the four groups. Samples marked by an asterisk (*) indicate significant differences (*p* <0.05) among the four groups. Samples marked with different lowercase letters indicate significant differences (a > b > c; *p* < 0.05) among the four groups.

Within our current investigation, we identified 32 digestion-related pathways, encompassing carbohydrates, glycans, proteins, amino acids, energy metabolism, and lipid metabolism. Among these, a total of 23 pathways displayed notable variations in abundance among the four groups (Figure 7). Interestingly, certain pathways associated with energy metabolism, such as carbon fixation in photosynthetic organisms, were found to be enriched in the NTL group. On the other hand, nitrogen metabolism exhibited enrichment in the BAL and BAS groups. Some of the amino acid metabolism pathways (i.e., lysine, phenylalanine, tryptophan, and tyrosine metabolism) were highly abundant in the BAL and NTS groups, whereas pathways related to glycolysis/gluconeogenesis, galactose, starch, and sucrose metabolism were more enriched in the NTL and BAS groups than in the NTS and BAL groups. Some lipid metabolites (glycerolipids, sphingolipids, glycerophospholipids, ether lipids, and alpha-linolenic metabolism) were more enriched in the NTL group, while fatty acid degradation and degradation of ketone bodies were more enriched in the BAL group.

## 4. Discussion

Investigating gut microbiomes can contribute to our understanding of host fitness and adaptation [36,37,38]. Previous studies have provided evidence that the gut microbiota of invasive species is linked to an increased capacity to colonize and thrive in new habitats [39,40]. Thus, exploring the gut microbiota of invasive fish species can provide effective information for elucidating the adaptive ability of invasive fish species. In the current study, the gut microbiome of NTS samples showed higher alpha diversity than BAS samples in similar habitats. The higher the alpha diversity of the gut microbiome, the more diverse food resources can be gained from the habitat. The microbiome that inhabits fish guts is metabolically versatile, a characteristic that can help the hosts expand their dietary options [17,37]. Moreover, complex gut microbiota can enhance the adaptability of the host to the environment and improve the successful invasion rate of alien species [41]. Invasive NT samples had a wider niche breadth than native BA samples. The niche width of microbiota is an important indicator of the effects of species sorting and dispersal restrictions on microorganisms [35]. The wider niche width of gut microbiota represented the higher metabolic flexibility and environmental adaptability of NT, suggesting that Nile tilapia can generally outcompete native species for food resources [1]. The gut microbiota in the BAS group had a narrower niche width than that of the BAL group, supporting the suggestion that dietary specialization in small juveniles of *M. terminals* was likely to be stronger than in sub-adult and adult groups [42]. In addition, we found that the proportion of generalists in NT was higher than in BA. Relevant research has defined generalists as microbes that inhabit a wide range of environments and display greater ecological adaptability, while specialists are defined as microbes that have a narrower range of occupancy across different environments [16]. In the present study, the gut microbial composition in juveniles and adults of NT and BA was compared at the phylum level. Proteobacteria, Firmicutes, and Fusobacteriota were the dominant phyla in the guts of NT and BA. Comparing our results with other studies, we found that the dominant microbes were quite similar among fish species at the phylum level [43,44]. A high abundance of *Cetobacterium* was observed in the NT. Bereded [45] also found that the dominant genus was *Cetobacterium* in the Nile Tilapia of Lake Tana and Lake Chamo. Some related studies have shown that the growth environment is a vital factor for gut microbial diversity [39,46]. However, the host’s dietary strategy was also regarded as a factor influencing the gut microbiome [9,47].

The gut microbiota can be regarded as a complex ecosystem [48,49,50]. In the microbial ecosystem, interactions between species are vital determinants of community structure and ecosystem function [51,52]. In this study, we found that microbial ecological networks in the NTS, NTL, BAS, and BAL groups were different. The gut microbial ecological networks showed remarkable variability in both interspecific and intraspecific individual comparisons. We found that cooperative interactions predominated in the NTL and NTS networks, whereas many competitive interactions were observed in the BAL and BAS networks, suggesting that the microbial ecological networks were associated with host species [53]. Relevant research has shown that facilitation and low levels of interspecies competition between these core microbes, potentially due to resource partitioning, contribute to the ecological adaptability of fish hosts [16]. In addition, previous studies have already provided evidence that the stability of the gut microbiota structure in fish can lead to the development of advantageous physiological functions, facilitate the utilization of novel ecological niches, and contribute to the formation of reproductive barriers. As a result, these factors can influence processes such as speciation or the expansion of the population range [11,54,55].

Functional analysis of the gut microbiota indicated that pathways related to glycolysis/gluconeogenesis, galactose, starch, and sucrose metabolism were more enriched in the BAS group than in the BAL group. Lysine, phenylalanine, tryptophan, and tyrosine metabolites were more abundant in the BAL group than in the BAS group (Figure 7). Our previous study indicated that the intestinal food items of *M. terminalis* in the developmental process were a transition phase in relation to a dietary shift and illustrated the food preferences between adults and juveniles [42]. Moreover, shifts in dietary preference were observed during *M. terminalis* growth, where juveniles preferred to consume phytoplankton and adults consumed more animal material (Xia et al., 2021) [42]. Carbon fixation in photosynthetic organisms, glycolysis/gluconeogenesis, galactose, and starch and sucrose metabolism were more enriched in the NTL group than in the BAL group, suggesting that adult Nile tilapia had a greater digestive ability for planktonic food items [56,57].

## 5. Conclusions

Above all, poor fishery management, water quality degradation, and loss of biodiversity have all facilitated the invasion of tilapia [58,59]. Compared with native fish species, the gut microbiome of invasive Nile tilapia showed several special characteristics (high alpha diversity, low levels of interspecies competition in the gut microbiome, and potential advantages in food utilization) that would contribute to producing high diversity in the dietary niches and also benefit the survival and local adaptation of the hosts. Nevertheless, it is necessary to explore gut microbiota data from invasive fish to provide a potential understanding of the differences in diet and physiological behavior. Therefore, further investigation of the effect of the gut microbiota on population expansion and the invasion ecology of wild invasive fishes should be a focus of future studies.

## Figures and Tables

**Table 1:** Alpha diversity results from gut microbial communities pertaining to the four groups.

Estimators	BAS–Mean	BAS–sd	BAL–Mean	BAL–sd	NTS–Mean	NTS–sd	NTL–Mean	NTL–sd
Ace	29.98 ^b^	6.68	47.87 ^a^	5.193	40.86 ^a^	7.57	44.51 ^a^	5.09
Chao1	29 ^b^	4.13	47.75 ^a^	5.312	41.17 ^a^	6.59	44.48 ^a^	5.24
PD	3.854 ^b^	0.3967	4.658 ^a^	0.5548	4.027 ^a^	1.21	4.439 ^a^	0.7405
Shannon	0.9917 ^b^	0.1346	2.368 ^a^	0.474	2.056 ^a^	0.678	2.13 ^a^	0.4143

Different superscript letters indicate significant differences in different groups, *p* < 0.05.

**Table 2:** Ecological networks estimators of gut microbiota in the four groups.

Networks Estimators	BAS	BAL	NTS	NTL
Number of nodes	52	85	84	87
Number of edges	224	429	422	467
Avg. number of neighbors	8.615	10.313	10.048	10.736
Network diameter	6	7	8	8
Characteristics path length	1.849	2.862	2.212	2.747
Clustering coefficient	0.354	0.636	0.341	0.506
Network density	0.084	0.126	0.061	0.125

## Data Availability

All fastq files obtained from sequencing are publicly available from the NCBI Sequence Read Archive (SRA) database (https://www.ncbi.nlm.nih.gov/sra/PRJNA979974) (accessed on 5 June 2023).

## Supplementary Materials

The following supporting information can be downloaded at: https://www.mdpi.com/article/10.3390/ani13152413/s1, Table S1: Basic environmental information of sampling site.
