# Supplementary material for: Fish Gut Microbiome Analysis Provides Insight into Differences in Physiology and Behavior of Invasive Nile Tilapia and Indigenous Fish in a Large Subtropical River in China
Source: Animals (Basel). 2023 Jul 26;13(15):2413. doi: 10.3390/ani13152413 (PMC10417376; doi:10.3390/ani13152413)
Supplement: Supplementary file 1 [file animals-13-02413-s001.zip › animals-2489862-supplementary.pdf]

SUPPLEMENTARY TABLES

Table S1 Basic environmental information of sampling site

	Item	Mean±SD
Environmental information	Temperature (℃)	28.7±0.4
	Salinity (‰)	0.01±0.00
	pH	8.2±0.2
	DO (mg/L)	7.1±0.1
	TP (mg/L)	0.14±0.04
	TN (mg/L)	1.9±0.5
	COD_{Mn} (mg/L)	2.4±0.3

DO, dissolved oxygen; TN, total nitrogen; TP, total phosphorus; COD_{Mn}, chemical oxygen demand
